# Molecular genetic approaches to decrease the uncontrolled misincorporation of non-canonical branched chain amino acids into recombinant mini-proinsulin expressed in *Escherichia coli*

**DOI:** 10.1186/s12934-022-01756-x

**Published:** 2022-03-04

**Authors:** Ángel Córcoles García, Peter Hauptmann, Peter Neubauer

**Affiliations:** 1 Sanofi-Aventis Deutschland GmbH, 65929 Frankfurt, Germany; 2 Chair of Bioprocess Engineering, Department of Biotechnology, Faculty III Process Sciences, Technische Universität Berlin, 10623 Berlin, Germany

**Keywords:** Non-canonical branched chain amino acids, Genetic engineering, Strain screening, Mini-reactor, Norvaline, Norleucine, Fed-batch

## Abstract

The uncontrolled incorporation of non-canonical branched chain amino acids (ncBCAAs) such as norleucine, norvaline and β-methylnorleucine into recombinant proteins in *E. coli* production processes is a crucial problem in the pharmaceutical industry, since it can lead to the production of altered proteins with non-optimal characteristics. Despite various solutions, to date there are no engineered strains that exhibit a reduced accumulation of these ncBAAs. In this study, novel *E. coli* K-12 BW25113 strains with exogenous tunable expression of target genes of the BCAA biosynthetic pathway were developed. For this purpose, single gene knock-outs for *thrA*, *ilvA*, *leuA*, *ilvIH*, *ilvBN*, *ilvGM* and *ilvC* were complemented with plasmids containing the respective genes under control of an arabinose promoter. These clones were screened in a mL-bioreactor system in fed-batch mode under both standard cultivation conditions and with pyruvate pulses, and induction of a min-proinsulin. Screening was performed by comparing the impurity profile of the recombinant mini-proinsulin expressed of each clone with the *E. coli* BW25113 WT strain, and the most promising clones were cultivated in a 15L Screening showed that up-regulation of *ilvC*, *ilvIH* and *ilvGM,* and downregulation of *leuA* and *ilvBN* trigger a reduction of norvaline and norleucine accumulation and misincorporation into mini-proinsulin. The stirred tank bioreactor cultivations confirmed that up-regulation of *ilvIH* and *ilvGM* were most effective to reduce the ncBCAA misincorporation. This novel approach for a reduced ncBCAA misincorporation may be solution to this old challenging problem in the large-scale production of human therapeutics.

**Supplementary Information:**

The online version contains supplementary material available at 10.1186/s12934-022-01756-x.

## Introduction

**Fig. 1:**
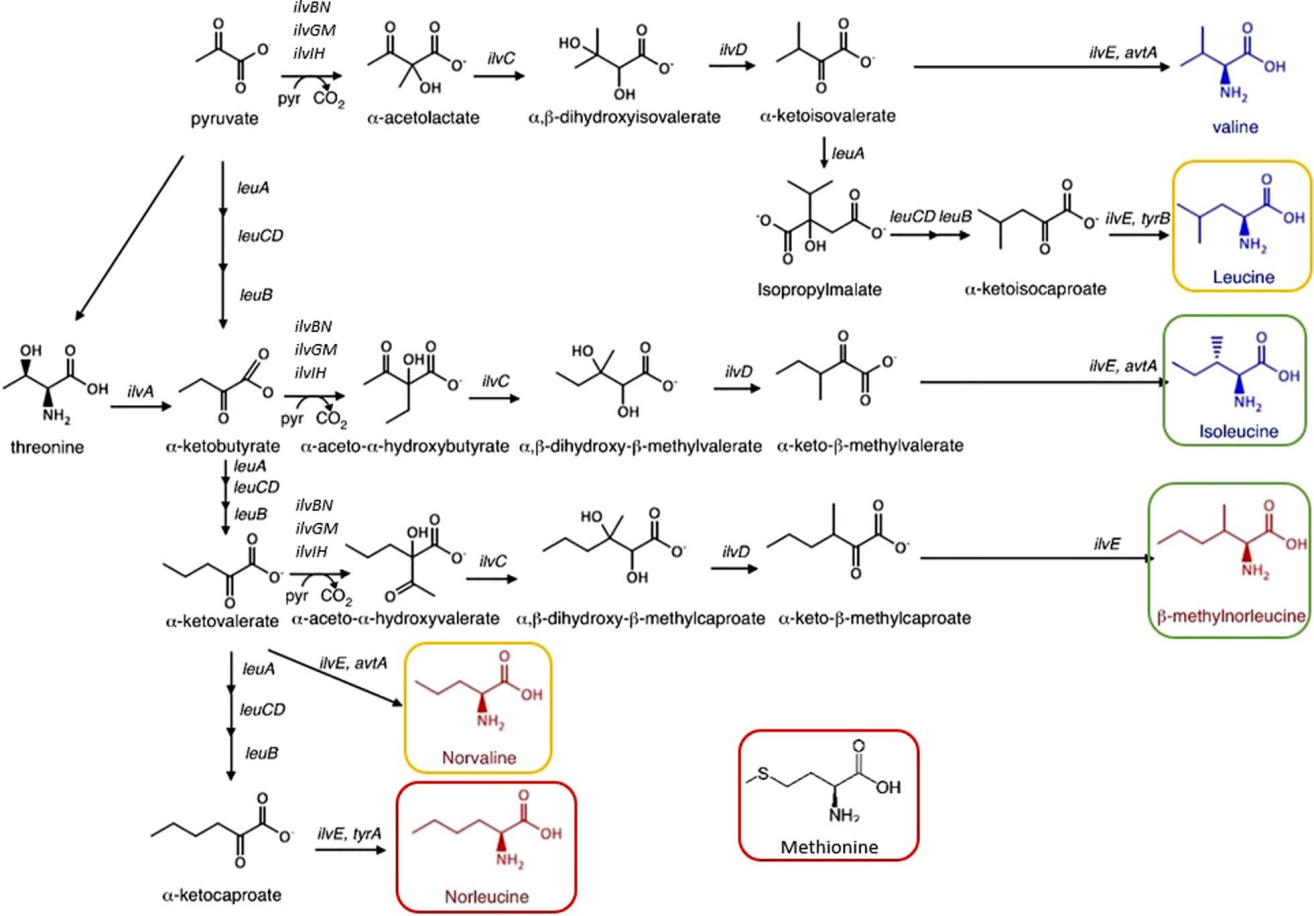
Schematic representation of the BCAA biosynthetic pathway. Molecular structure of canonical BCAAs appear in blue while non-canonical BCAAs are shown in red. The corresponding canonical/non-canonical amino acid pairs are framed in the same color, e.g. pair leucine/norvaline is framed in yellow. See main text for detailed description. Adapted from Reitz et al. [24]

Biosynthesis and misincorporation of non-canonical branched chain amino acids (ncBCAAs) such as norleucine, norvaline and β-methylnorleucine into recombinant proteins has been reported in a number of *E. coli* production processes. NcBCAAs can be synthesized by *E. coli* metabolism as by-products of the BCAA biosynthetic pathway as a result of the low specificity of the *leu* and *ilv*-operon-encoded enzymes for their substrates. There are three main factors triggering ncBCAA biosynthesis in *E. coli*: overflow metabolism driven by glucose accumulation and oxygen limitation due to inefficient mixing in large-scale reactors [1, 2], de-regulation of enzymes encoded by the *leu* operon resulting from leucine depletion—particularly during production of leucine-rich recombinant proteins [3]—and genetic background of the *E. coli* strain used as host [4]. Free ncBCAA can be mis-incorporated into cellular proteins through tRNA mis-aminoacylation during protein translation due to their structural similarity with the respective canonical amino acids. For instance, leucyl-tRNA synthetase (leuRS) can transfer both leucine and the non-canonical counterpart norvaline, which only differ by a single methyl group [5]. Similarly, methionyl-tRNA synthetase (metRS) can catalyze methionine and norleucine [6], and isoleucyl-tRNA synthetase (ileRS), isoleucine and β-methylnorleucine [7] (Fig. 1). Such misincorporation might lead to the production of altered recombinant proteins, having non optimal characteristics e.g. altered biological activity, modulated sensitivity to proteolysis and immunogenicity [8]. This represents an important concern for the pharmaceutical industry since product quality is pivotal for recombinant proteins that are to be used as human therapeutics in order to ensure patient safety. Several valuable strategies for reducing misincorporation of ncBCAAs into recombinant proteins have been already described in the literature: mutation of methionine codons of the gene encoding the recombinant protein [9], co-expression of enzymes capable of degrading ncBCAAs [10], supplementation of exogenous canonical amino acids [11], overproduction of methionine by mutating genes involved in methionine and threonine biosynthesis [12, 13], knocking-out genes involved in the BCAA biosynthetic pathway [3, 14, 15], optimization of fermentation conditions [4], supplementation of trace elements [16] and use of alternative *E. coli* expression strains with a genetic background less prone to non-canonical BCAA misincorporation [4]. However, most of the aforementioned strategies have numerous limitations, which impede them to be effectively applied in large-scale recombinant protein production processes. This study focuses on another approach which has not yet been explored and that might contribute to close the scientific gap; engineering of novel *E. coli* strains allowing tunable expression of target genes involved in the BCAA biosynthetic pathway to reduce the biosynthesis of the ncBCAAs.

The first substrate of both isoleucine and ncBCAA biosynthetic pathways is α-ketobutyrate, which can be either synthesized from threonine by threonine deaminase (*ilvA*), or from pyruvate via keto acid chain elongation by the leucine operon encoded enzymes (*leuABCD*) [15]. As mentioned before, the synthesis and intracellular accumulation of ncBCAAs results from the substrate promiscuity of the *leu* and *ilv*-operon encoded enzymes, involved in the BCAA biosynthetic pathway. This explains the sequential keto acid chain elongation from pyruvate to α-ketocaproate over α-ketobutyrate and α-ketovalerate by the actuation of the *leu* enzymes α-isopropylmalate synthase (*leuA*), β-isoprolylmalate dehydrogenase (*leuB*) and α-isoprolylmalate isomerase (*leuCD*). Despite α-ketoisovalerate is the intermediate for biosynthesis of canonical BCAAs valine and leucine and thus the preferred substrate for α-isopropylmalate synthase, this enzyme also shows certain affinity towards alternative α-keto acids such as pyruvate, α-ketobutyrate and α-ketovalerate [17–20]. The transformation of the two last ones by the *leu*-operon encoded enzymes generates intermediates for the formation of the ncBAAs. Moreover, the three consecutive reactions catalyzed by the *ilv*-operon encoded enzymes acetohydroxy acid synthase (*ilvBN*, *ilvGM*, *ilvIH*), acetohydroxy acid isomeroreductase (*ilvC*) and dihydroxyacid dehydratase (*ilvD*) are involved not only in the biosynthetic pathways of valine and isoleucine, but also in the synthesis pathway of β-methylnorleucine. Acetohydroxy acid synthase (AHAS) catalyzes a 2-step enzymatic reaction. In the first reaction step only pyruvate can bind to AHAS, catalyzing decarboxylation to form an active intermediate. In the second reaction step three alternative substrates (pyruvate, α-ketobutyrate or α-ketovalerate) can bind to the pre-formed intermediate to generate three alternative acetohydroxy acids. Pyruvate is the preferred substrate for acetohydroxy acid synthase, but it is also able to transform the other two. Nevertheless, additionally it must be considered that there are three different isoenzymes of acetohydroxy acid synthase, each one showing different substrate preference and catalytic properties [21, 22]. Also transaminase B (*ilvE*) shows substrate promiscuity since it catalyzes transamination of leucine, valine, isoleucine, norleucine, norvaline, β-methylnorvaline and homoisoleucine intermediates [23]. A schematic representation of the ncBCAA biosynthetic pathway is shown in Fig. 1.

The BCAA biosynthesis pathway is effectively modulated by a complex regulatory network. This enables cells to adapt BCAA biosynthesis to shifting environmental conditions. Regulation can take place at enzymatic or genetic level (for a compact overview see Additional file 1: Table S1). At post-translational level, many of the target genes are subjected to feedback inhibition and/or allosteric activation. Aspartokinase/homoserine dehydrogenase (*thrA*) is inhibited by threonine and activated by isoleucine and methionine [25]. Threonine deaminase (*ilvA*) is inhibited by isoleucine and activated by valine [26, 27]. Acetohydroxy acid synthase isozyme III (*ilvIH*) is feedback-inhibited by valine, leucine and isoleucine [28]. α-isopropylmalate synthase (*leuA*) is inhibited by leucine [29], while acetohydroxy acid synthase isozyme I is inhibited by valine [28]. Furthermore, transcription of operons *ilvBN*, *ilvGMEDA*, *leuABCD* and *thrABC* is regulated by attenuation [30]. Operon *ilvYC* is regulated by an operon-specific mechanism mediated by protein IlvY. In addition, operons *ilvIH, ilvGMEDA* and *leuABCD* are regulation by Lrp (leucine-responsive protein). *ilvBN* operon is subjected to global catabolite repression control mediated by CAP (catabolite activator protein) as well. Moreover, the global IHF (integration host factor) also regulates expression of operons *ilvBN* and *ilvGMEDA* [28]. Additionally, the global RelA/SpoT modulon is also described to regulate transcription of operons implicated in amino acid biosynthesis [31].

To decrease ncBCAA biosynthesis, the following strategies could be effective by modulating the expression of different genes of the pathway: (i) limit conversion of pyruvate to α-ketobutyrate, (ii) limit transformation of threonine to α-ketobutyrate and (iii) limit conversion of α-ketobutyrate to α-ketovalerate. Strategy (i) might be achieved by down-regulating operon *leuABCD* but also by up-regulating operon *ilvBN*, strategy (ii) may be realized by down-regulating the *thr* genes as well as *ilvA*. Strategy (iii) could be accomplished by down-regulating the expression of *leuABCD* and up-regulating *ilvIH*, *ilvGM* or *ilvC.* According to this hypothesis novel *E. coli* strain mutants were genetically engineered so that the expression of single target genes (*leuA*, *thrA*, *ilvA*, *ilvC*, *ilvIH*, *ilvBN* and *ilvGM*) could be modulated in order to evaluate the effect of genetic modulation in ncBCAA biosynthesis.

We used the *araBAD* promoter in order to regulate expression of the target genes. In order to ensure functionality of the *araBAD* promoter, strain *E. coli* K-12 BW25113 was selected as the model organism for this study, since it is deficient in arabinose catabolizing enzymes. Furthermore, in order to properly evaluate the effect that expression regulation of target genes has on ncBCAA biosynthesis and subsequent misincorporation into cellular proteins, mini-proinsulin was selected as a model recombinant protein in this study. The protein sequence of recombinant mini-proinsulin contains several canonical amino acids (3 methionine, 14 leucine and 5 isoleucine residues) that can be potentially substituted by the non-canonical counterparts (norleucine, norvaline and β-methylnorlelucine, respectively) upon mistranslation. In order to regulate expression of target genes involved in the BCAA biosynthetic pathway it was first necessary to eliminate endogenous expression of such genes in the *E. coli* cell. Hence, following single target genes/operons were knocked out from the *E. coli* K-12 BW25113 genome by homologous recombination: *thrA*, *ilvA*, *leuA*, *ilvIH*, *ilvBN*, *ilvGM* and *ilvC*. Expression regulation of previously knocked-out genes was carried out by transforming arabinose-based tunable expression plasmids (pACG_araBAD series) containing the native sequence of the target genes into the respective engineered *E. coli* K-12 BW25113 KO mutants. Plasmid pSW3_*lacI*^+^ expressing recombinant mini-proinsulin was additionally transformed into the aforementioned mutants in order to evaluate effect of genetic regulation on ncBCAA misincorporation. The engineered tunable *E. coli* mutants were screened in a mini-reactor by triggering induction of different expression levels of the target genes. The impurity profile of recombinant mini-proinsulin was then compared with the control non-engineered *E. coli* host. Screening was performed in fed-batch mode under standard cultivation conditions and under conditions mimicking large-scale effects i. e. pyruvate pulsing and DO limitation [32]. After screening in the mini-reactor system, the most promising candidate strains were cultivated in fed-batch cultivations in a 15 L bioreactor and the impurity profile of the recombinant protein was analysed in order to confirm the improvement.

## Materials and methods

### Strains and strain engineering

*E. coli* K-12 BW25113 [F^−^ DE(*araD-araB*)567 *lacZ4787(*del)::*rrnB-3* LAM^−^ *rph-1* DE(*rhaD-rhaB*)568 *hsdR*514] was used as reference strain. A library of knock-out (KO) strains derived from the reference strain was generated by homologous recombination, according to the method described by Datsenko and Wanner [33]. The KO strains consisted of single knock-outs of genes involved in the branched-chain amino acid biosynthesis pathway: *leuA*, *thrA*, *ilvA*, *ilvC*, *ilvIH*, *ilvBN* or *ilvGM*. In order to modulate expression of the aforementioned knocked-out genes, arabinose-based tunable expression plasmids carrying the native sequence of the corresponding target gene (Additional file 1: Figure S1, pACG_araBAD plasmid series) were transformed into the KO strains. All strains used in this study contained additionally the plasmid pSW3_*lacI*^+^s (Additional file 1: Figure S2) for expression of recombinant mini-proinsulin (MPI) under the control of an isopropyl-β-D-thiogalactopyranosid (IPTG)-inducible *tac*-promoter.

### Cultivation medium

Cultivations were performed in mineral salt medium containing (per L): 2.0 g Na_2_SO_4_, 2.468 g (NH_4_)_2_SO_4_, 0.5 g NH_4_Cl, 14.6 g K_2_HPO_4_, 3.6 g NaH_2_PO_4_ × 2 H_2_O and 1.0 g (NH_4_)2-H-citrate, 2 mL of a 1.0 M MgSO_4_ solution, and 2 mL of a trace elements solution. The trace elements solution contained (per L): 0.5 g CaCl_2_ × 2 H_2_O, 0.18 g ZnSO_4_ × 7 H_2_O, 0.1 g MnSO_4_ × H_2_O, 16.7 g FeCl_3_ × 6 H_2_O, 0.16 g CuSO_4_ × 5 H_2_O and 0.18 g CoCl_2_ × 6 H_2_O.The medium contained the carbon source as described in the sections below. All chemicals were from Sigma-Aldrich (Munich, Germany) with exception of NH_4_Cl and MnSO_4_ × H_2_O, which were from Merck (Darmstadt, Germany).

### Evaluation of L-arabinose induction of gene expression in E. coli mutants

The aim of these experiments was to evaluate the effect of different L-arabinose concentrations on the expression of target genes involved in the BCAA biosynthetic pathway, which are under the control of an *araBAD* promoter in the mutant *E. coli* strains engineered in this study.

Cultures were prepared by inoculating 50 μL of a cryostock of the corresponding *E. coli* strain into 15 mL tubes pre-loaded with 5 mL of 1:3 diluted supplemented mineral salt medium containing 5 g L^−1^ glucose (Merck, Darmstadt, Germany), 0.1 M Na-phosphate buffer (Merck, Darmstadt, Germany) and 100 µg mL^−1^ ampicillin (Sigma-Aldrich, Munich, Germany). For the tunable *E.coli* strains, medium also contained 25 µg mL^−1^ chloramphenicol (Sigma-Aldrich, Munich, Germany). Different concentrations of L-arabinose were added to the different cultures prepared for each strain and these were incubated at 37 °C and 250 rpm, overnight. OD_600_ was then measured after a cultivation time of 16 h. Results are reported in Additional file 1: Figure S3.

### Cultivation conditions in the PALL24 mini-reactor system

In this study, two different cultivation modes were tested in a Pall Micro24 reactor system (Microreactor Technologies Inc., Mountain View, CA, USA), as described in our previous paper [32]. The first is the reference cultivation and consists of a glucose-limited fed-batch cultivation under aerobic conditions. The second cultivation type is a glucose-limited fed-batch cultivation where pyruvate pulses and transient down-shifts of the oxygen supply were applied additionally to trigger DO limitation.

Cultivation conditions for the reference strain were described in García et al. [32]. However, additional considerations were taken into account in this study to adapt the method to the tunable *E. coli* strains, which are described below.

Cultivation medium of pre-cultures for the tunable *E.coli* strains also contained 25 µg mL^−1^ chloramphenicol (Sigma-Aldrich, Munich, Germany) and the minimum L-arabinose (Sigma-Aldrich, Munich, Germany) concentration necessary to recover cell growth levels of the reference *E. coli* K-12 BW25113 pSW3_*lacI*^+^ strain (Additional file 1: Figure S3): 0.05% L-arabinose for *ilvA* tunable *E. coli*, 0.1% for *leuA* tunable *E. coli*, 0.4% for *ilvC* and *thrA* tunable *E. coli* and 0% for *ilvBN*-, *ilvIH* and *ilvGM* tunable *E.coli* strains. In addition, cultivation medium for the tunable *E. coli* strains in the STR was supplemented with different concentrations of L-arabinose in order to trigger different expression levels of the target gene in the tunable *E. coli* strains: 0.05/0.2/0.8% L-arabinose for *ilvA*, *ilvBN*, *ilvGM* and *ilvIH* tunable *E. coli*, 0.1/0.2/0.4% for *leuA* tunable *E. coli* and 0.4/0.8/1.6% for *ilvC* and *thrA* tunable *E. coli* strains.

### Cultivation conditions in the 15L STR

As for the PALL24 mini-reactor system, two different cultivation conditions were tested in a 15 L stirred-tank reactor. The experimental procedure for the reference strain is described in the Materials and Methods section in García et al. [32]. However, following points were additionally considered in this experiment in order to adapt the method to the tunable *E. coli* strains.

Cultivation medium in pre-cultures, feed solution and STR for *ilvIH* and *ilvGM* tunable *E. coli* strains additionally contained 25 µg mL^−1^ chloramphenicol. Moreover, reactor medium also contained 0.8% L-arabinose from the beginning for expression induction of target genes.

### Analysis of cell growth and mini-proinsulin

Cell growth was determined by the optical density at 600 nm (OD_600_) in an Ultraspec 2100 pro photometer (Amersham Bioscience, Marlborough, MA, USA). If necessary, samples were diluted with the original medium into an OD_600_ range of 0.3–0.8.

Concentration of recombinant mini-proinsulin was determined from hourly samples taken after IPTG induction from the *E. coli* cultivations carried out in the STR according to an HPLC method internally available at Sanofi-Aventis Deutschland GmbH.

### Analysis of ncBCAAs

NcBCAA analysis was performed as previously described by García et al. [32]. NcBCAA concentrations were analysed from hourly samples taken after IPTG induction from *E. coli* cultivations performed in the STR. NcBCAA concentrations from cultivations carried out in the mini-reactor system were only determined at the end of the cultivation (2–3.5 h after induction, depending on the tested strain) due to the limited available volumes.

## Results

### Screening of tunable E. coli strains in a PALL24 mini-reactor system, based on ncBCAA misincorporation into recombinant mini-proinsulin

Previously [32] we demonstrated that by combining the enzyme-based glucose delivery method Enbase® in a PALL24 mini-bioreactor system with repeated pyruvate pulses and simultaneous oxygen downshifts, an increase in the ncBCAAs norvaline and norleucine in both intracellular soluble protein and recombinant mini-proinsulin inclusion body fractions occurs. This cultivation strategy is easy to apply under standard laboratory conditions for strain screening purposes since it might facilitate identification of *E. coli* strains with a phenotype more resistant to ncBCAA misincorporation, which are more valuable for recombinant protein manufacturing.

**Fig. 2:**
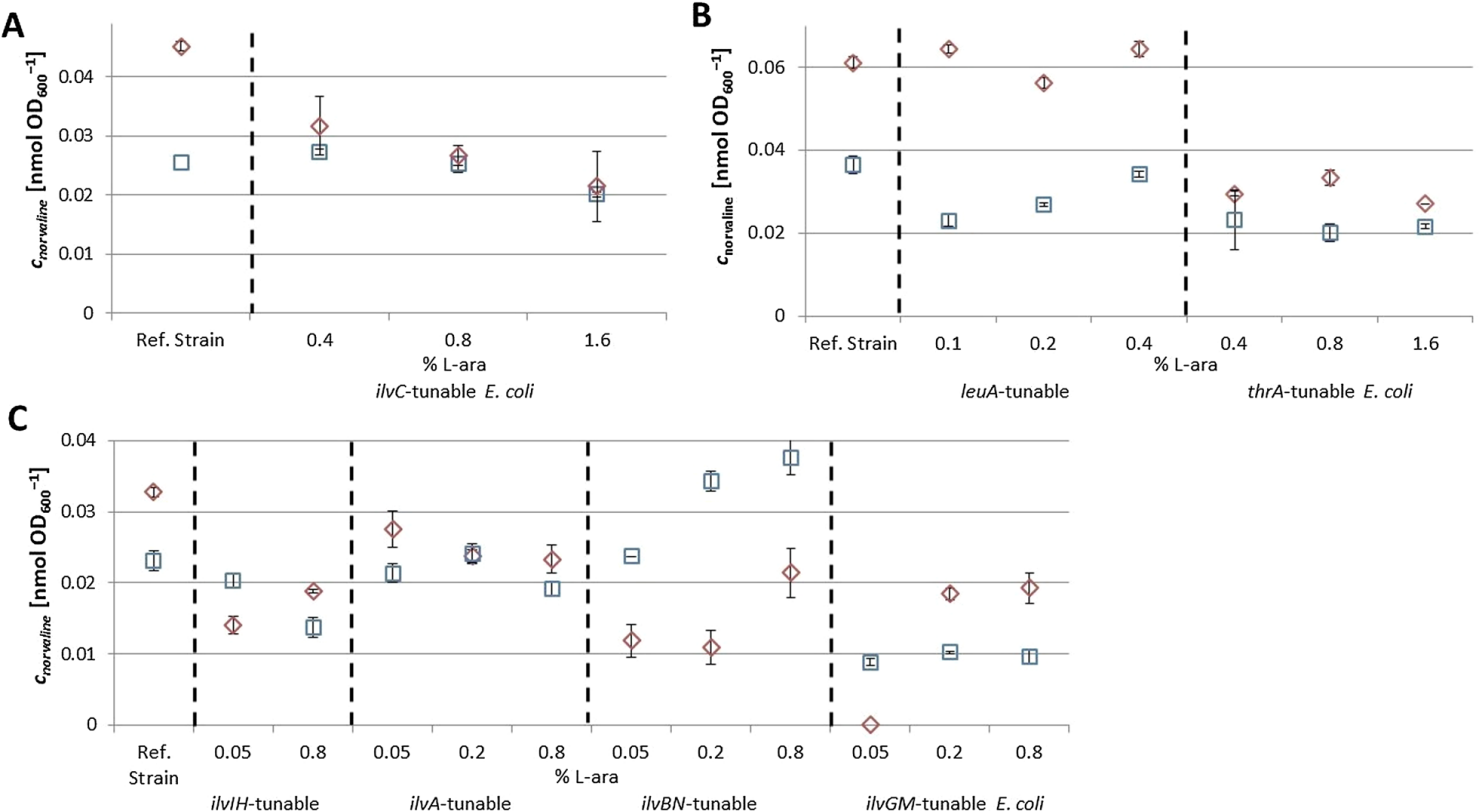
Molar concentrations of norvaline normalized to OD_600_ in the inclusion body fraction from samples taken from glucose-limited fed-batch cultivations 2 h after IPTG induction of *ilvC* tunable *E. coli* (**A**), 3.5 h after IPTG induction of *leuA* and *thrA* tunable *E. coli* strains (**B**) and 3 h after induction of *ilvIH*, *ilvA*, *ilvBN* and *ilvGM* tunable *E. coli* strains (**C**) in a 10 mL PALL24 mini-reactor with different L-arabinose concentrations and cultivation modes. Two cultivation modes were tested: reference cultivation () and cultivation under simultaneous pyruvate pulsing and dissolved oxygen (DO) limitation (◇). Strain *E. coli* K-12 BW25113 pSW3_*lacI*^+^ (named “wild type *E. coli”* in the chart) was employed as the reference strain. Results represent the average of 3 technical replicates

In the current study, several *E. coli* strains with controllable expression of target genes involved in the BCAA biosynthetic pathway (*leuA*, *thrA*, *ilvA*, *ilvC*, *ilvIH*, *ilvBN* and *ilvGM*) were cultivated in parallel in a PALL24 mini-bioreactor system in fed-batch mode under reference cultivation conditions and under conditions reproducing large-scale effects. In order to assess the impact of gene regulation on the misincorporation of ncBCAAs norvaline, norleucine und ß-methylnorleucine into recombinant mini-proinsulin, different concentrations of L-arabinose were tested for each strain, which trigger different expression levels of the target gene. Screening was performed by comparing the impurity profile of the recombinant mini-proinsulin expressed in each tested strain with the non-engineered *E. coli* BW25113 host. For each cultivation, molar concentrations of norvaline present in the recombinant mini-proinsulin inclusion body fraction were analyzed from samples taken 2 to 3.5 h after IPTG induction, depending on the tested *E. coli* strain (Fig. 2). An overview of the experimental design is shown in Additional file 1: Tables S2 and S3.

In the reference cultivation, *ilvC* and *ilvIH tunable E. coli* reported a significant decrease of norvaline concentration in the inclusion body fraction when adding increasing concentrations of L-arabinose into the medium. The opposite behavior was observed for *ilvBN* tunable *E. coli*. For *thrA* and *ilvGM tunable E. coli* strains*,* norvaline concentration showed a significant reduction with respect to the control *E. coli* strain but effect of increasing L-arabinose concentrations did not show a clear effect on ncBCAA concentration. For *ilvA tunable E. coli* no significant variation of norvaline concentration was reported with respect to the control *E. coli* strain and effect of increasing L-arabinose concentrations did not seem to show a clear effect on ncBCAA concentration. The highest reduction of norvaline concentration with respect to the non-engineered *E. coli* BW25113 host was reported when inducing *ilvIH tunable E. coli* with 0.8% L-arabinose (-40.7%) and *ilvGM* tunable *E. coli* with 0.05% L-arabinose (-61.8%)*.* In the cultivation with pyruvate pulses combined with DO limitation, all tested strains with exception of *leuA* and *ilvIH tunable E. coli* showed the same pattern described at the previous paragraph for the reference cultivation. Interestingly, the two aforementioned strains reported the opposite effect (Fig. 2). Results for norleucine are in accordance with norvaline (see Additional file 1: Figure S4)

### Verification of ilvGM and ilvIH tunable E. coli strains in a 15 L bioreactor under cultivation conditions reproducing large-scale effects

**Fig. 3:**
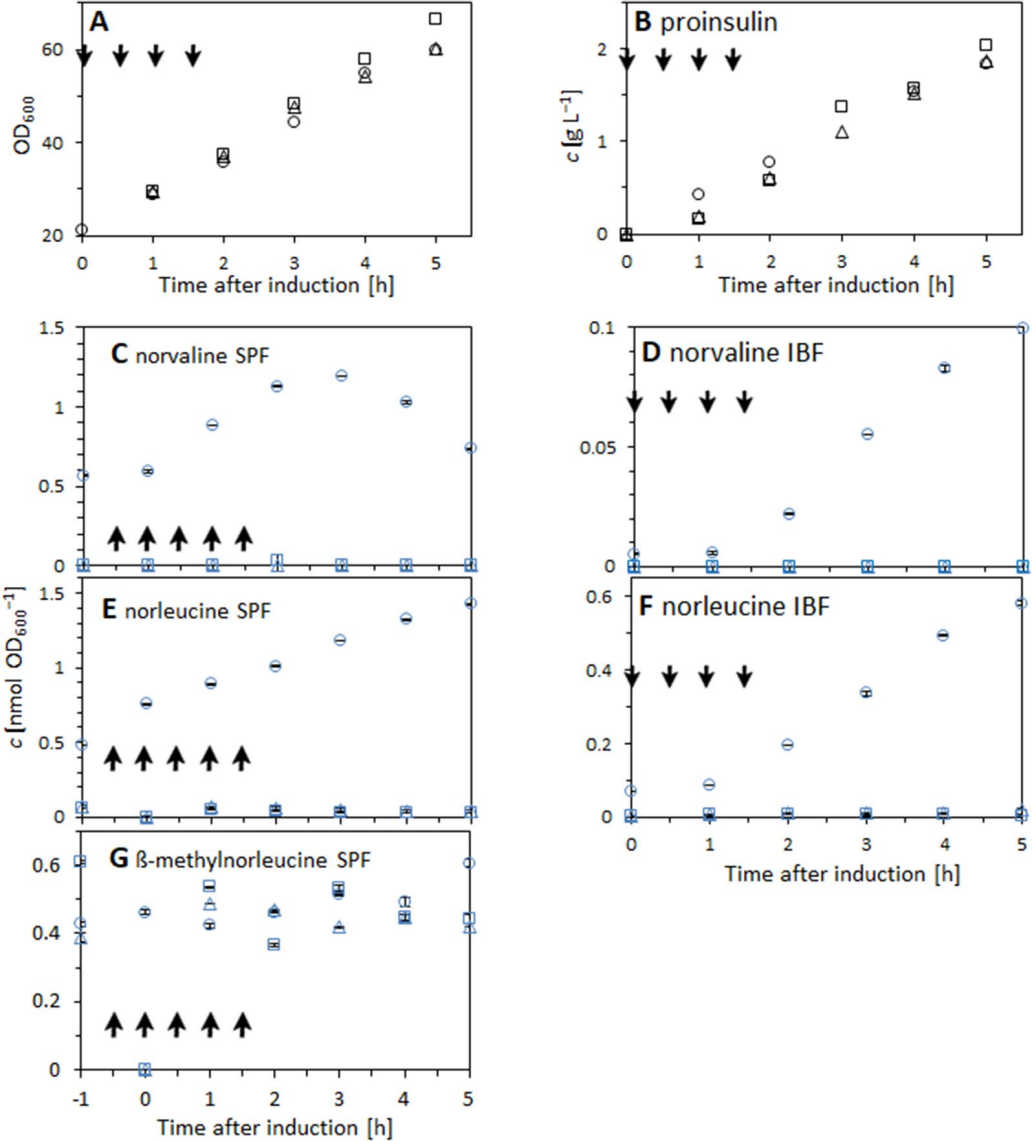
Growth and mini-proinsulin production in 15 L stirred tank bioreactor fed-batch cultivations with conditions triggering ncBCAA accumulation (i.e. pyruvate pulsing combined with DO limitation as described in the Materials and Methods section) of different tunable and reference E. coli strains, and accumulation of ncBCAAs in the intracellular soluble protein fraction (ISPF) and in the inclusion bodies. **A** OD_600_, **B** volumetric concentration of mini-proinsulin (**B**), molar concentrations of norvaline and norleucine normalized to OD_600_ in the ISPF (**C**, **E**) and inclusion body fraction (**D**, **F**), and molar concentrations of ß-methylnorleucine normalized to OD_600_ present in the ISPF (**G**). Samples were analyzed hourly after IPTG induction. —Reference cultivation with *E. coli* K-12 BW25113 pSW3_*lacI*^+^; □—*ilvGM* tunable strain *E. coli* K-12 BW25113 pSW3_*lacI*^+^ pACG_araBAD_*ilvGM*; Δ—*ilvIH* tunable strain *E. coli* K-12 BW25113 Δ*ilvIH* pSW3_*lacI*^+^ pACG_araBAD_*ilvIH*. Arrows indicate time points where 1 g L^−1^ pyruvate pulses combined with 5 min DO limitation were applied. Results represent the average of 3 technical replicates. Results for the reference strain were already published in García et al. [32]

According to screening results shown in previous section, *ilvIH* and *ilvGM* tunable *E. coli* strains induced with 0.8% L-arabinose showed the highest reduction of ncBCAA misincorporation into recombinant mini-proinsulin, compared to the non-engineered strain. The aim of the current experiment was to verify the performance of the aforementioned potential tunable *E. coli* strains in a 15 L stirred-tank reactor in fed-batch mode and under cultivation conditions triggering formation of ncBCAAs, i.e. pyruvate pulses and DO limitation, in order to confirm their advantage as strains ensuring recombinant product quality. The impurity profile of the recombinant mini-proinsulin expressed by each tested strain was compared with the non-engineered *E. coli* BW25113 reference strain over cultivation time. For each cultivation, molar concentrations of norvaline, norleucine and ß-methylnorleucine present in the intracellular soluble protein fraction and in the recombinant mini-proinsulin inclusion body fraction were analyzed from hourly samples taken after IPTG induction. In addition, OD_600_ and recombinant mini-proinsulin concentration were analyzed over cultivation time for each tested strain (Fig. 3). Results reported in Fig. 3 for the reference strain were already published by García et al. [32]. In order to avoid catabolite repression and enable arabinose induction of target genes, glucose levels remained limiting during cultivation, according to fed-batch cultivation approach used in this study (data not shown).

The cultivation of the control *E. coli* strain subjected to pyruvate pulses combined with DO limitation showed a progressive accumulation of norleucine and β-methylnorleucine in the intracellular soluble protein fraction over time after induction, being most significant for norleucine. Furthermore, norvaline concentration also increased progressively under the aforementioned cultivation conditions, but only until 3 h after induction. From that time point on, norvaline concentration decreased until reaching initial values at 5 h after induction. This might suggest that after 2 h, i.e. after the last pyruvate pulse combined with DO limitation, its associated effect triggering norvaline accumulation is not active anymore. Both tested potential mutants in cultivations, the *ilvGM* and *ilvIH* tunable strains showed much lower levels of norvaline and norleucine concentrations in the intracellular soluble protein fraction. The level was lowest in the *ilvGM* tunable strain. However, β-methylnorleucine concentrations did not significantly vary with respect to the control cultivation. It is noteworthy to highlight, that for most samples norvaline could not be properly detected as its concentration was below the limit of detection (Fig. 3, Charts C, E and G).

The cultivation of the control *E. coli* strain subjected to pyruvate pulses combined with DO limitation showed a progressive accumulation of norvaline and norleucine in the inclusion body fraction over time after induction. Both tested potential mutants in cultivations with the *ilvGM* and *ilvIH* tunable *E. coli* showed a dramatic reduction of norvaline and norleucine concentrations in the inclusion bodies, and this decrease was more pronounced for norleucine in the *ilvGM* tunable strain. Norvaline could not be detected in any case for both tested mutants (Fig. 3, Charts D and F).

Moreover, the effect caused by pyruvate pulses with the concomitant DO limitation on growth (OD_600_) and on intracellular accumulation of recombinant mini-proinsulin was really similar in any of the tested strains. Hence, the specific production of the recombinant mini-proinsulin by the *ilvIH* and *ilvGM* tunable *E. coli* strains is at least as good as by the reference strain (Fig. 3, Charts A and B).

## Discussion

In this study, novel *E. coli* strain mutants were genetically engineered so that expression of single target genes (*leuA*, *thrA*, *ilvA*, *ilvC*, *ilvIH*, *ilvBN* and *ilvGM*) could be modulated in order to evaluate its effect on ncBCAA biosynthesis and misincorporation into recombinant mini-proinsulin. Screening of the engineered *E. coli* mutants was performed in a 10 mL mini-reactor under reference cultivation conditions. Additional screening of mutants under cultivation conditions triggering ncBCAA, i. e. pyruvate pulses and DO limitation, allowed elucidating performance of mentioned *E. coli* mutants under cultivation conditions which were earlier shown to enhance the production of the ncBCAAs. In order to support discussion of the reported results following hypothesis already described in literature were assumed: (i) since the recombinant protein expressed in this study has a high leucine-content, a depletion of the intracellular leucine pool after IPTG induction is expected, thus causing de-regulation of the *leu* operon, which, in turn, triggers a relative increase of ncBCAA biosynthesis [5, 15], (ii) under cultivation conditions based on pyruvate pulses and DO limitation an intracellular accumulation of pyruvate and, consequently, an increase of the metabolic flux through leucine, valine and ncBCAA pathways is expected [1], I). In addition, for each scenario, transcriptional and post-translational regulation of target genes was also taken into consideration for discussion.

Down-regulation of *leuA* was hypothesized to limit ncBCAA biosynthesis by restricting the successive keto acid chain elongation reactions starting from pyruvate until ncBCAA formation. Some investigations previously demonstrated that knocking-out one or more *leu* genes reduces ncBCAA biosynthesis [3, 15]. Results obtained for *leuA* tunable *E. coli* under standard cultivation conditions in this study are in accordance with the logic of the metabolic pathway since, for the mentioned *E. coli* mutant, norvaline and norleucine concentrations present in both tested protein fractions progressively increased by adding increasing concentrations of L-arabinose into the medium, i.e. by increasing *leuA* expression. Interestingly, under cultivation conditions subjected to pyruvate pulses and DO limitation, increasing *leuA* expression did not translate into significant variation of ncBCAA concentrations but those remained always higher than under standard cultivation conditions. This observation might be explained due to a saturation of the metabolic pathway comprising the consecutive keto acid chain elongation reactions starting from pyruvate and leading to ncBCAA caused by the expected intracellular accumulation of pyruvate and de-regulation of *leu* under such cultivation conditions, so that higher *leuA* expression would then not trigger more carbon flux to ncBCAA formation.

Up-regulation of *ilvC* was hypothesized to trigger reduction of ncBCAA biosynthesis, since that would stimulate the metabolic flux from α-ketobutyrate through the isoleucine biosynthetic pathway, thereby relatively reducing α-ketobutyrate availability for ncBCAA formation. The results confirmed the hypothesis since, for *ilvC* tunable *E. coli* under standard cultivation conditions, norvaline and norleucine concentrations present in both tested protein fractions progressively decreased by adding increasing concentrations of L-arabinose into the medium, i.e. by increasing *ilvC* expression. The same trend was observed when pyruvate pulsing and DO limitation conditions were applied. As expected, under those cultivation conditions, ncBCAA concentrations were higher.

AHAS III has more substrate preference for α-ketobutyrate than pyruvate [22, 28, 34]. Hence, an increase in AHAS III concentration would favor the metabolic flux from α-ketobutyrate into α-acetohydroxy-butyrate at the expense of the alternative enzymatic reaction leading to ncBCAA synthesis. Results confirmed this hypothesis since for *ilvIH* tunable *E. coli* under standard cultivation conditions, norvaline and norleucine concentrations present in both tested protein fractions progressively decreased by adding increasing concentrations of L-arabinose into the medium, i. e. by increasing *ilvIH* expression. Surprisingly, under cultivation conditions subjected to pyruvate pulses and DO limitation, the opposite trend was observed: increasing *ilvIH* expression was translated into an increase of ncBCAA concentrations. The simultaneous presence of multiple factors playing a role in the regulation of the BCAA metabolic pathway under mentioned cultivation conditions made challenging to find a plausible explanation for those observations.

The *ilvGM* tunable *E. coli* strain also reported a reduction of norvaline and norleucine concentrations compared with the wild type *E. coli* strain under both tested cultivation conditions. As expected, under cultivation conditions subjected to pyruvate pulsing and DO limitation, ncBCAA concentrations were higher. Moreover, the highest ncBCAA reduction observed among all tested mutant strains was reported for this mutant. This might be because AHAS II shows the highest substrate preference for α-ketobutyrate among the AHAS isozymes, being then most of the metabolic flux from α-ketobutyrate directed to the isoleucine pathway. In addition, *k*_*cat*_/*K*_*m*_ of AHAS II is about 20-fold higher than AHAS III [22, 28, 34]. However, increasing of *ilvGM* expression did not translate into significant variation of ncBCAA concentrations. That might be explained by a negative feedback regulation due to an excess of free isoleucine, which would decrease AHAS II activity [28], thus counteracting the increased AHAS II activity driven by L-arabinose addition. Thus, in the ideal case, *ilvGM* expression should be up-regulated as much as possible but without leading to an accumulation of isoleucine.

Unexpectedly, the *ilvBN tunable E. coli* strain showed the opposite behavior than the tested *ilvIH*- and *ilvGM* tunable *E. coli* strains under standard cultivation conditions: norvaline and norleucine concentrations progressively increased by adding increasing concentrations of L-arabinose into the medium, i. e. by increasing *ilvBN* expression. As opposed to AHAS II and III, AHAS I prefers pyruvate to α-ketobutyrate as substrate [22, 28, 34]. Hence, an increase in AHAS I concentration would favor the metabolic flux from pyruvate into the valine and leucine biosynthetic pathway at the expense of pyruvate transformation to α-ketobutyrate by the *leu* operon. However, up-regulation of *ilvBN* might trigger overproduction of valine, which is demonstrated to inhibit enzymatic activity of AHAS III by feedback regulation and to activate enzymatic activity of L-threonine dehydratase (*ilvA*-encoded enzyme) [28]. According to this configuration, more α-ketobutyrate would be produced from the threonine pathway and, since activity of AHAS III is feedback regulated, α-ketobutyrate might then preferably enter the ncBCAA biosynthetic pathway to the detriment of the isoleucine biosynthetic pathway. This metabolic configuration might explain why, as opposed to first hypothesized, a down-regulation of operon *ilvBN* triggers reduction of ncBCAA biosynthesis. The same trend was observed when pyruvate pulsing and DO limitation conditions were applied but, interestingly, ncBCAA concentrations were lower than under standard conditions. The simultaneous presence of multiple factors playing a role in the regulation of the BCAA metabolic pathway under mentioned cultivation conditions made challenging to find a plausible explanation for those observations.

The *thrA tunable E. coli* strain showed a reduction of norvaline and norleucine concentrations in both tested protein fractions compared with the wild type strain under both tested cultivation conditions. As expected, under cultivation conditions subjected to pyruvate pulsing and DO limitation, ncBCAA concentrations were higher. However, effect of increasing L-arabinose concentrations did not show a clear effect on ncBCAA concentrations. With 0.4% L-ara, a significant decrease in norvaline and norleucine was observed. However, a further increase in *thrA* expression when using higher L-ara concentrations did not show any further variation of ncBCAA concentration if compared with 0.4% L-ara. That might be explained by a bottleneck taking place in the metabolic pathway downstream of *thrA*, so that a higher *thrA* expression does not translate in higher α-ketobutyrate production. In addition, it is well known that L-threonine allosterically inhibits enzyme activity of *thrA*-encoded enzyme [25] so that an excessive arabinose induction triggers accumulation of threonine, then causing feedback inhibition of enzymatic activity, hence annulling the higher expected *thrA* expression. This observation might indicate that the threonine metabolic pathway is not the one redirecting more flux to α-ketobutyrate but the pyruvate pathway.

The *ilvA* tunable *E. coli* strain showed that, mainly in the intracellular protein soluble protein fraction, norvaline and norleucine concentrations report a reduction compared with the reference strain under both tested cultivation conditions but effect of increasing L-arabinose concentrations did not show a clear trend on variation of ncBCAA concentrations. As expected, under cultivation conditions subjected to pyruvate pulsing and DO limitation, ncBCAA concentrations were higher. With 0.05% L-ara, a significant decrease in norvaline and norleucine levels was observed. However, a further increase in *ilvA* expression when using higher L-ara concentrations did not show any further reduction of ncBCAA concentration if compared with 0.05% L-ara. As for *thrA*, that might be explained by a bottleneck in the metabolic pathway downstream of *ilvA.* Moreover, it is well known that isoleucine allosterically inhibits the enzyme activity of L-threonine dehydratase (*ilvA)* [26, 27]. We suggest that a strong induction with arabinose triggers accumulation of isoleucine, then causing feedback inhibition of enzymatic activity, hence annulling the higher expected *ilvA* expression. As stated before, this observation might indicate that the threonine metabolic pathway is not the one redirecting more flux to α-ketobutyrate but the pyruvate pathway.

The positive results with the *ilvIH* and *ilvGM* tunable *E. coli* strains could be successfully confirmed in the 15 L reactor. These novel *E. coli* strains showing a strongly reduced ncBCAA misincorporation profile may be useful in the pharmaceutical industry. The use of these strains as standard hosts for recombinant protein production might result in a higher manufacturing efficiency as well as an excellent product quality, which is crucial for recombinant proteins intended for human therapeutics.

## Conclusions and outlook

This study was aimed to close the scientific gap by developing a new scientific approach in order to reduce ncBCAA biosynthesis and subsequent misincorporation into recombinant proteins since all the alternative strategies published so far had numerous disadvantages which challenge their effective application. In this study we demonstrated that ncBCAA biosynthesis can be exogenously controlled by fine tuning expression of target genes involved in the BCAA biosynthetic pathway. By screening different engineered tunable *E. coli* mutants in a mini-reactor system we demonstrated that an up-regulation of *ilvC*, *ilvIH* and *ilvGM* and down-regulation of *leuA* and *ilvBN* trigger a reduction of norvaline and norleucine biosynthesis and misincorporation into recombinant mini-proinsulin expressed in this study. Concerning target genes *ilvA* and *thrA*, results support previous results which show that the threonine pathway is not providing the highest metabolic flux to α-ketobutyrate. Among the tested genes, up-regulation of *ilvIH* and *ilvGM* showed the highest reduction of ncBCAA biosynthesis and misincorporation.

Interestingly, norleucine was the most mis-incorporated ncBCAA and β-methylnorleucine levels did not significantly change under the tested experimental conditions, which may suggest that β-methylnorleucine is synthesized by an alternative unknown metabolic pathway. Potential *ilvIH* and *ilvGM* tunable *E. coli* strains showing a preferred protein impurity profile during screening in the mini-reactor system were further verified in a 15 L reactor under cultivation conditions subjected to pyruvate pulsing and DO limitation, with the same recombinant mini-proinsulin production and a highly significant reduction of ncBCAA misincorporation into the recombinant protein in comparison with the non-engineered *E. coli* strain. This is in accordance with what was reported at mini-reactor level, thus confirming the reliability and robustness of the aforementioned strains. These novel *E. coli* strains might then be employed as expression hosts in large-scale reactors for industrial production of recombinant proteins with a reduced ncBCAA misincorporation profile. Testing of the engineered *E. coli* strains in alternative scale-down reactors such as a 2-compartment scale-down reactor [35] would give more hints about their applicability in industrial scale recombinant protein production processes.

Moreover, optimal expression levels of single target genes leading to a reduction of ncBCAA misincorporation into recombinant mini-proinsulin were investigated in this current study. However, it might be also very interesting to investigate expression regulation of two or more target genes simultaneously in order to achieve further reduction of ncBCAA misincorporation that might not be possible by just considering a single gene. Recommended genetic combinations which probably would provide the lowest production and incorporation of ncBCAAs are: (i) down-regulation of *leuA* and repair or up-regulation of operon *ilvGM*, (ii) down-regulation of *leuA* and up-regulation of the *ilvIH* operon. These combinations may be with other methods such as: (i) avoidance of codons in the sequence gene of interest that cause a higher ncBCAA misincorporation, (ii) protein engineering to increase specificity of biosynthetic enzymes for certain α-keto acids, and (iii) protein engineering to increase specificity of aminoacyl tRNA synthetases for the canonical amino acids.

## Supplementary Information

**Additional file 1: Table S1**. Summary of the regulation mechanisms affecting the target genes investigated in this study. **Figure S1**. Genetic map of pACG_araBAD plasmid variants generated in 637 this study: empty pACG_araBAD (a), pACG_araBAD_ilvA (b), pACG_araBAD_thrA (c), pACG_araBAD_leuA (d), pACG_araBAD_ilvBN (e), pACG_araBAD_ilvGM (f), pACG_araBAD_ilvIH (g) and pACG_araBAD_ilvC (h). Plasmid maps were generated by Snapgene®. **Figure S2**. Genetic map of plasmid pSW3_lacI+. Plasmid map was generated by Snapgene®. **Figure S3**. OD600 measured 16h after cultivation of* E. coli* mutant strains under different L-arabinose concentrations. The reference strain* E. coli* K-12 BW25113 pSW3_lacI+ is also included as a control for comparison. **Figure S4**. Molar concentrations of norleucine normalized to OD600 in the inclusion body fraction from samples taken from glucose-limited fed-batch cultivations 2 h after IPTG induction of ilvC tunable* E. coli* (A), 3.5 h after IPTG induction of leuA and thrA tunable* E. coli* strains (B) and 3 h after induction of ilvIH, ilvA, ilvBN and ilvGM tunable* E. coli* strains (C) in a 10 mL PALL24 mini-reactor with different L-arabi nose concentrations and cultivation modes. Two cultivation modes were tested: reference cultivation (□) and cultivation under simultaneous pyruvate pulsing and dissolved oxygen (DO) limitation (◇).Strain E. coli K-12 BW25113 pSW3_lacI+ (named “wild type* E. coli*” in the chart) was employed as the reference strain. Results represent the average of 3 technical replicates. **Table S1**. Overview of the different cultivation conditions tested in each well of the first mini-reactor plate with the reference strain* E. coli* K-12 BW25113 pSW3_lacI+, and the tunable strains for leuA, ilvC and thrA. **Table S22**. Overview of the different cultivations conditions tested in each well of the second mini-re actor plate with the reference strain E. coli BW25113 pSW3_lacI+, and the tunalbe strains for ilvIH, ilvA, ilvBN and ilvGM.

## References

[1] Soini J, Falschlehner C, Liedert C, Bernhardt J, Vuoristo J, Neubauer P. Norvaline is accumulated after a down-shift of oxygen in Escherichia coli W3110. Microb Cell Fact. 2008; 7: 1–14. (I) 10.1186/1475-2859-7-30 PMC2579280 18940002

[2] Soini J, Ukkonen K, Neubauer P (2011). Accumulation of amino acids deriving from pyruvate in *Escherichia coli* W3110 during fed-batch cultivation in a two-compartment scale-down bioreactor. Adv Biosci Biotechnol.

[3] Fenton D, Lai PH, Lu H, Mann M, Tsai L. Control of norleucine incorporation into recombinant proteins. US Patent 5599690. 1994

[4] Ni J, Gao M, James A, Yao J, Yuan T, Carpick B (2015). Investigation into the misincorporation of norleucine into a recombinant protein vaccine candidate. J Ind Microbiol Biotechnol.

[5] Apostol I, Levine J, Lippincott J, Leach J, Hess E, Glascock CB (1997). Incorporation of norvaline at leucine positions in recombinant human hemoglobin expressed in *Escherichia coli*. J Biol Chem.

[6] Kiick KL, Weberskirch R, Tirrell DA (2001). Identification of an expanded set of translationally active methionine analogues in *Escherichia coli*. FEBS Lett.

[7] Muramatsu R, Misawa S, Hayashi H (2003). Finding of an isoleucine derivative of a recombinant protein for pharmaceutical use. J Pharm Biomed Anal.

[8] Laird MW, Veeravalli K. Methods and compositions for preventing norleucine misincorporation into proteins. US Patent Application WO 2014/047311 A1. 2013.

[9] Brunner et al. Fermentation media and methods for controlling norleucine in polypeptides. US Pat. No. 5698418. 1997.

[10] Bogosian G, O’Neil JP, Smith HQ. Prevention of incorporation of non-standard amino acids into proteins. US Patent 8603781. 2013.

[11] Abu-Absi N, Inlow D, Macdonald AC, Poulhzan M. Preventing norvaline and norleucine misincorporation in recombinant proteins. US Patent Application WO 2007/103521 A3. 2008.

[12] Usuda Y, Kurahashi O (2005). Effects of deregulation of methionine biosynthesis on methionine excretion in *Escherichia coli*. Appl Environ Microbiol.

[13] Veeravalli K, Laird MW, Fedesco M, Zhang Y, Yu XC (2015). Strain engineering to prevent norleucine incorporation during recombinant protein production in *Escherichia coli*. Biotechnol Prog.

[14] Tsai LB (1988). Control of misincorporation of de novo synthesized norleucine into recombinant interleukin-2 in *E. coli*. Biochem Biophys Res Commun.

[15] Bogosian G, Violand BN, Dorward-King EJ, Workman WE, Jung PE, Kane JF (1989). Biosynthesis and incorporation into protein of norleucine by *Escherichia coli*. J Biol Chem.

[16] Biermann M, Linnemann J, Knüpfer U, Vollstädt S, Bardl B, Seidel G, Horn U (2013). Trace element associated reduction of norleucine and norvaline accumulation during oxygen limitation in a recombinant Escherichia coli fermentation. Microb Cell Fact.

[17] Hunter MF, Parker EJ (2014). Modifying the determinants of α-ketoacid substrate selectivity in mycobacterium tuberculosis α-isopropylmalate synthase. FEBS Lett.

[18] Wiegel J, Schlegel HG (1977). α-Isopropylmalate synthase from Alcaligenes eutrophus H 16. Arch Microbiol.

[19] Kisumi M, Sugiura M, Chibata I. Biosynthesis of norvaline, norleucine, and homoisoleucine in Serratia marcescens. J Biochem 1976; 80(2): 333–339. (I) 10.1093/oxfordjournals.jbchem.a131281 794063

[20] Kohlhaw G, Leary TR, Umbarger HE (1969). α-Isopropylmalate synthase from Salmonella typhimurium purification and properties. J Biol Chem.

[21] Gollop N, Damri B, Barak ZE, Chipman DM (1989). Kinetics and mechanism of acetohydroxy acid synthase isozyme III from *Escherichia coli*. Biochemistry.

[22] Vinogradov V, Vyazmensky M, Engel S, Belenky I, Kaplun A, Kryukov O (2006). Acetohydroxyacid synthase isozyme I from Escherichia coli has unique catalytic and regulatory properties. Biochimica et Biophysica Acta (BBA)-General Subjects.

[23] Yu X, Wang X, Engel PC (2014). The specificity and kinetic mechanism of branched-chain amino acid aminotransferase from *Escherichia coli* studied with a new improved coupled assay procedure and the enzyme's potential for biocatalysis. FEBS J.

[24] Reitz C, Fan Q, Neubauer P (2018). Synthesis of non-canonical branched-chain amino acids in *Escherichia coli* and approaches to avoid their incorporation into recombinant proteins. Curr Opin Biotechnol.

[25] Patte JC, Neidhardt FC, Curtiss R, Ingraham JL, Lin ECC, Low KB, Magasanik B, Reznikoff WS, Riley M, Schaechter M, Umbarger HE (1996). Biosynthesis of threonine and lysine. *Escherichia coli* and Salmonella: cellular and molecular biology.

[26] Umbarger HE. Evidence for a negative-feedback mechanism in the biosynthesis of isoleucine. American Association for the Advancement of Science; 1956. 10.1126/science.123.3202.848 13324101

[27] Monod J, Wyman J, Changeux JP (1965). On the nature of allosteric transitions: a plausible model. J Mol Biol.

[28] Salmon KA, Yang CR, Hatfield GW. Biosynthesis and regulation of the branched-chain amino acids†. EcoSal Plus 2006; 2(1). 10.1128/ecosalplus.3.6.1.5 26443574

[29] Soper TS, Doellgast GJ, Kohlhaw GB (1976). Mechanism of feedback inhibition by leucine: purification and properties of a feedback-resistant α-isopropylmalate synthase. Arch Biochem Biophys.

[30] Vitreschak AG, Lyubetskaya EV, Shirshin MA, Gelfand MS, Lyubetsky VA (2006). Attenuation regulation of amino acid biosynthetic operons in proteobacteria: comparative genomics analysis. FEMS Microbiol Lett.

[31] Fang M, Bauer CE (2018). Regulation of stringent factor by branched-chain amino acids. Proc Natl Acad Sci.

[32] García ÁC, Hauptmann P, Neubauer P (2021). Glucose-limited fed-batch cultivation strategy to mimic large-scale effects in *Escherichia coli* linked to accumulation of non-canonical branched-chain amino acids by combination of pyruvate pulses and dissolved oxygen limitation. Microorganisms.

[33] Datsenko KA, Wanner BL (2000). One-step inactivation of chromosomal genes in *Escherichia coli* K12 using PCR products. Proc Natl Acad Sci.

[34] Barak Z, Chipman DM, Gollop NATAN (1987). Physiological implications of the specificity of acetohydroxy acid synthase isozymes of enteric bacteria. J Bacteriol.

[35] Limberg MH, Pooth V, Wiechert W, Oldiges M (2016). Plug flow versus stirred tank reactor flow characteristics in two-compartment scale-down bioreactor: Setup-specific influence on the metabolic phenotype and bioprocess performance of Corynebacterium glutamicum. Eng Life Sci.

