# Supplementary material for: Molecular genetic approaches to decrease the uncontrolled misincorporation of non-canonical branched chain amino acids into recombinant mini-proinsulin expressed in Escherichia coli
Source: Microb Cell Fact. 2022 Mar 4;21:30. doi: 10.1186/s12934-022-01756-x (PMC8896088; doi:10.1186/s12934-022-01756-x)
Supplement: Supplementary file 1 — Additional file 1: Table S1. Summary of the regulation mechanisms affecting the target genes investigated in this study. Figure S1. Genetic map of pACG_araBAD plasmid variants generated in 637 this study: empty pACG_araBAD (a), pACG_araBAD_ilvA (b), pACG_araBAD_thrA (c), pACG_araBAD_leuA (d), pACG_araBAD_ilvBN (e), pACG_araBAD_ilvGM (f), pACG_araBAD_ilvIH (g) and pACG_araBAD_ilvC (h). Plasmid maps were generated by Snapgene®. Figure S2. Genetic map of plasmid pSW3_lacI+. Plasmid map was generated by Snapgene®. Figure S3. OD600 measured 16h after cultivation of E. coli mutant strains under different L-arabinose concentrations. The reference strain E. coli K-12 BW25113 pSW3_lacI+ is also included as a control for comparison. Figure S4. Molar concentrations of norleucine normalized to OD600 in the inclusion body fraction from samples taken from glucose-limited fed-batch cultivations 2 h after IPTG induction of ilvC tunable E. coli (A), 3.5 h after IPTG induction of leuA and thrA tunable E. coli strains (B) and 3 h after induction of ilvIH, ilvA, ilvBN and ilvGM tunable E. coli strains (C) in a 10 mL PALL24 mini-reactor with different L-arabi nose concentrations and cultivation modes. Two cultivation modes were tested: reference cultivation (□) and cultivation under simultaneous pyruvate pulsing and dissolved oxygen (DO) limitation (◇).Strain E. coli K-12 BW25113 pSW3_lacI+ (named “wild type E. coli” in the chart) was employed as the reference strain. Results represent the average of 3 technical replicates. Table S1. Overview of the different cultivation conditions tested in each well of the first mini-reactor plate with the reference strain E. coli K-12 BW25113 pSW3_lacI+, and the tunable strains for leuA, ilvC and thrA. Table S22. Overview of the different cultivations conditions tested in each well of the second mini-re actor plate with the reference strain E. coli BW25113 pSW3_lacI+, and the tunalbe strains for ilvIH, ilvA, ilvBN and ilvGM. [file 12934_2022_1756_MOESM1_ESM.pdf]

Supplementary material

Table S1. Summary of the regulation mechanisms affecting the target genes investigated in this study.

Gene	Enzyme	Transciptional regulation	Post-translational regulation
leuA	2-isopropylmalate synthase	-Attenuation (by leu)<br>-Lrp regulation (leu does not show a modulation effect)<br>-RelA/SpoT modulon (up-regulation by (p)ppGpp after amino acid starvation)	-Feed-back inhibition (by leu)
ilvC	Ketol-acid reducto-isomerase (NADP(+))	-Substrate-mediated activation (substrate binding to a preformed IlvY protein DNA complex relaxes an IlvY protein-induced DNA bend and increases the affinity for RNA polymerase)<br>-RelA/SpoT modulon (up-regulation by (p)ppGpp after amino acid starvation)	-
ilvIH	acetohydroxyacid synthase isozyme III	-Lrp regulation (leu inhibits Lrp activator from binding to the promoter regulatory region of ilvIH operon thus decreasing transcription)<br>-RelA/SpoT modulon (down-regulation by (p)ppGpp after amino acid starvation)	-Feed-back inhibition (by val, leu and ile)
ilvGM	acetohydroxyacid synthase isozyme II	-Attenuation (val, leu and ile)<br>-Lrp regulation (leu inhibits Lrp repressor from binding to the transcription site of ilvGMEDA operon thus increasing transcription)<br>-IHF-mediated global regulation (activation)<br>-RelA/SpoT modulon (up-regulation by (p)ppGpp after amino acid starvation)	-
ilvBN	acetohydroxyacid synthase isozyme I	-Attenuation (by val and leu)<br>-CAP-mediated global regulation (activation when glc levels are low)<br>-IHF-mediated global regulation (activation)<br>-RelA/SpoT modulon (up-regulation by (p)ppGpp after amino acid starvation)	-Feed-back inhibition (by val)
thrA	threonine-sensitive, bifunctional aspartokinase/ homoserine dehydrogenase 1	-Attenuation (thr and ile)<br>-RelA/SpoT modulon (up-regulation by (p)ppGpp after amino acid starvation)	-Feed-back inhibition (thr).<br>-Activation by ile and met.
ilvA	L-threonine dehydratase	-<br>-	-Feed-back inhibition (ile). Activation by valine.

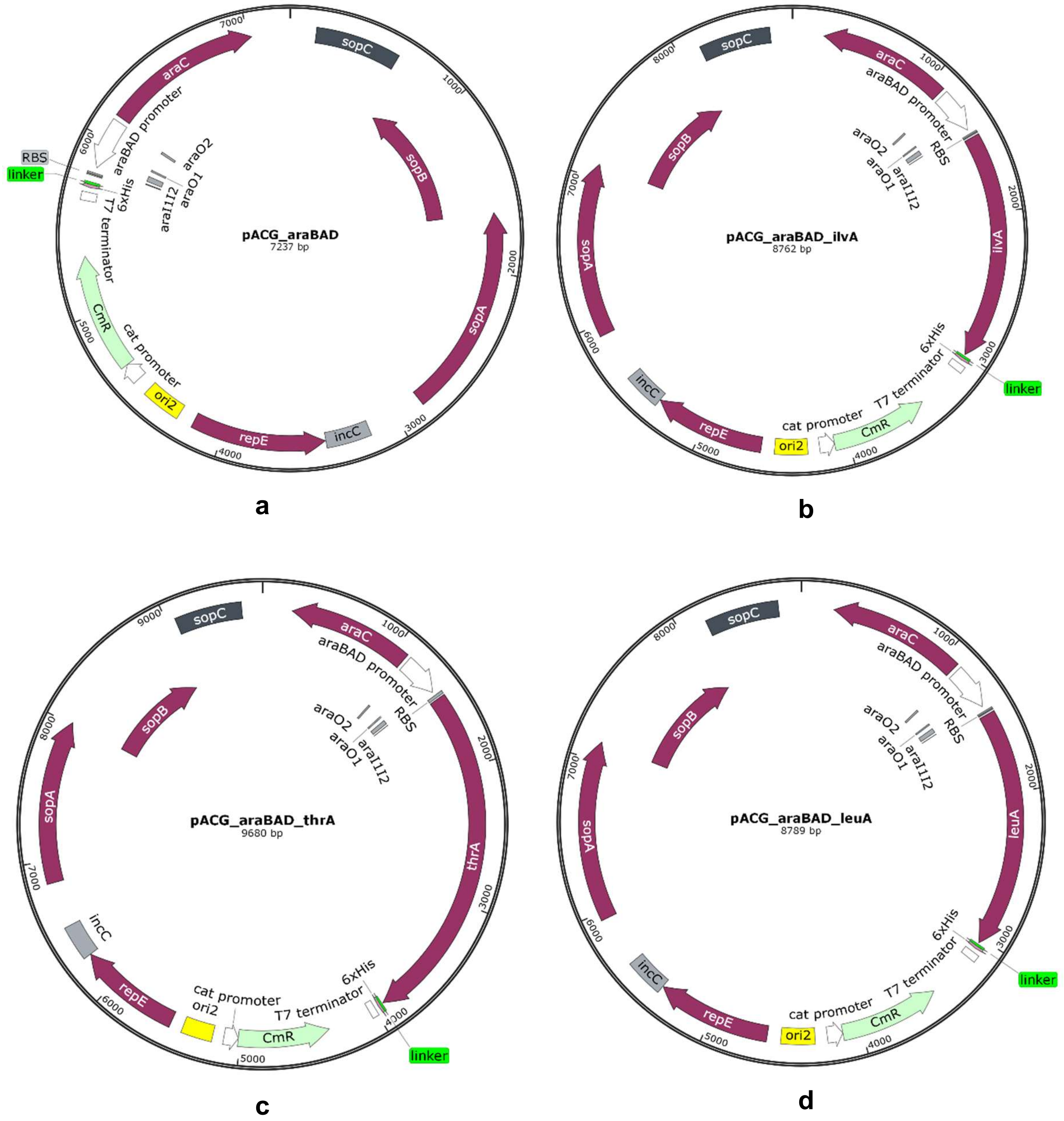
pACG_araBAD
7237 bp
pACG_araBAD_ilvA
8762 bp
pACG_araBAD_thrA
9680 bp
pACG_araBAD_leuA
8789 bp
araC
araBAD promoter
araO2
araO1
araI1I2
RBS
linker
6xHis
T7 terminator
CmR
cat promoter
ori2
repE
incC
sopA
sopB
sopC
ilvA
thrA
leuA
a
b
c
d

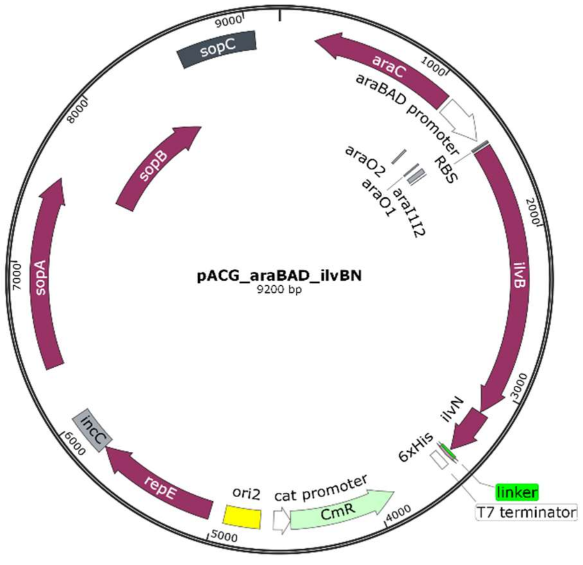

e

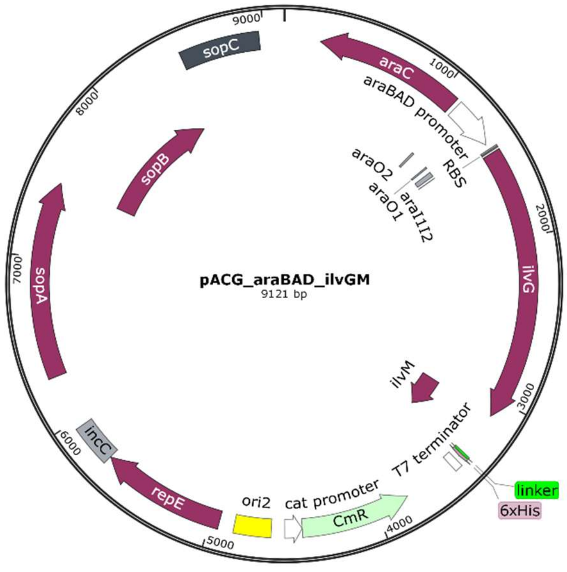

f

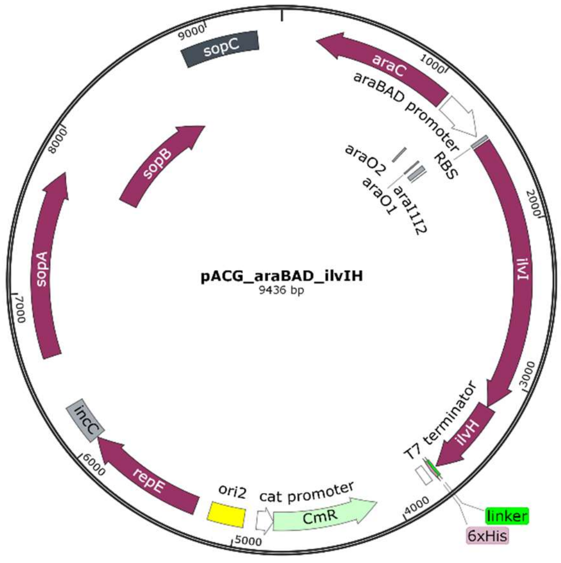

g

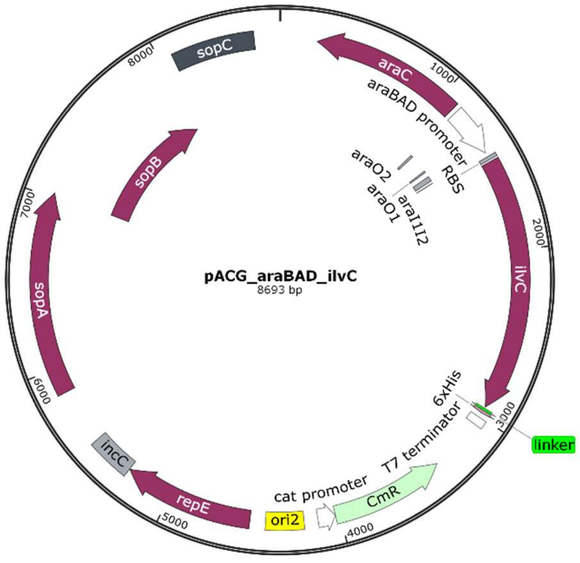

h

Figure S1. Genetic map of pACG_araBAD plasmid variants generated in this study: empty pACG_araBAD (a), pACG_araBAD_*ilvA* (b), pACG_araBAD_*thrA* (c), pACG_araBAD_*leuA* (d), pACG_araBAD_*ilvBN* (e), pACG_araBAD_*ilvGM* (f), pACG_araBAD_*ilvIH* (g) and pACG_araBAD_*ilvC* (h). Plasmid maps were generated by Snapgene®.

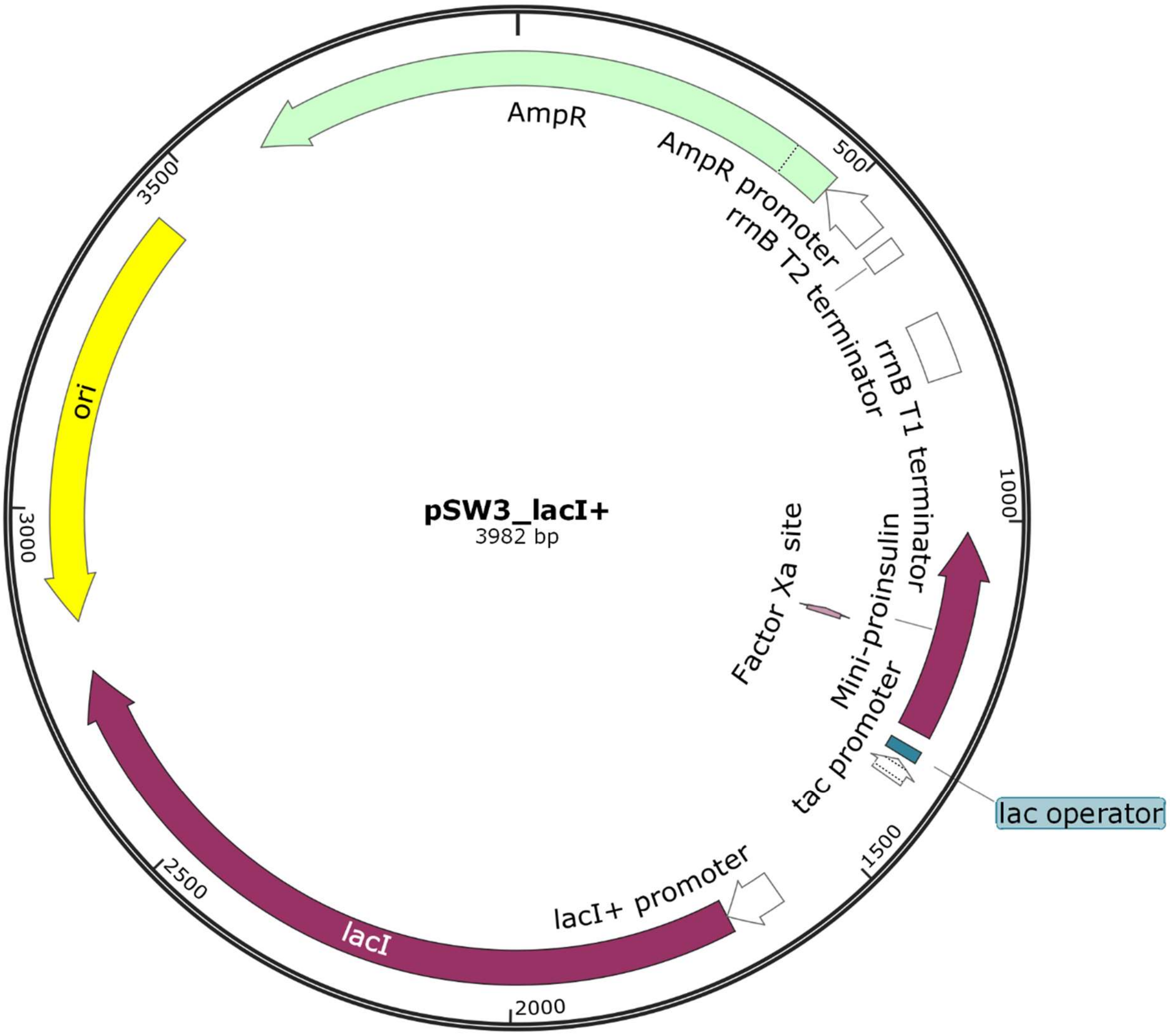

Figure S2. Genetic map of plasmid pSW3_*lacI*$^{+}$. Plasmid map was generated by Snapgene®.

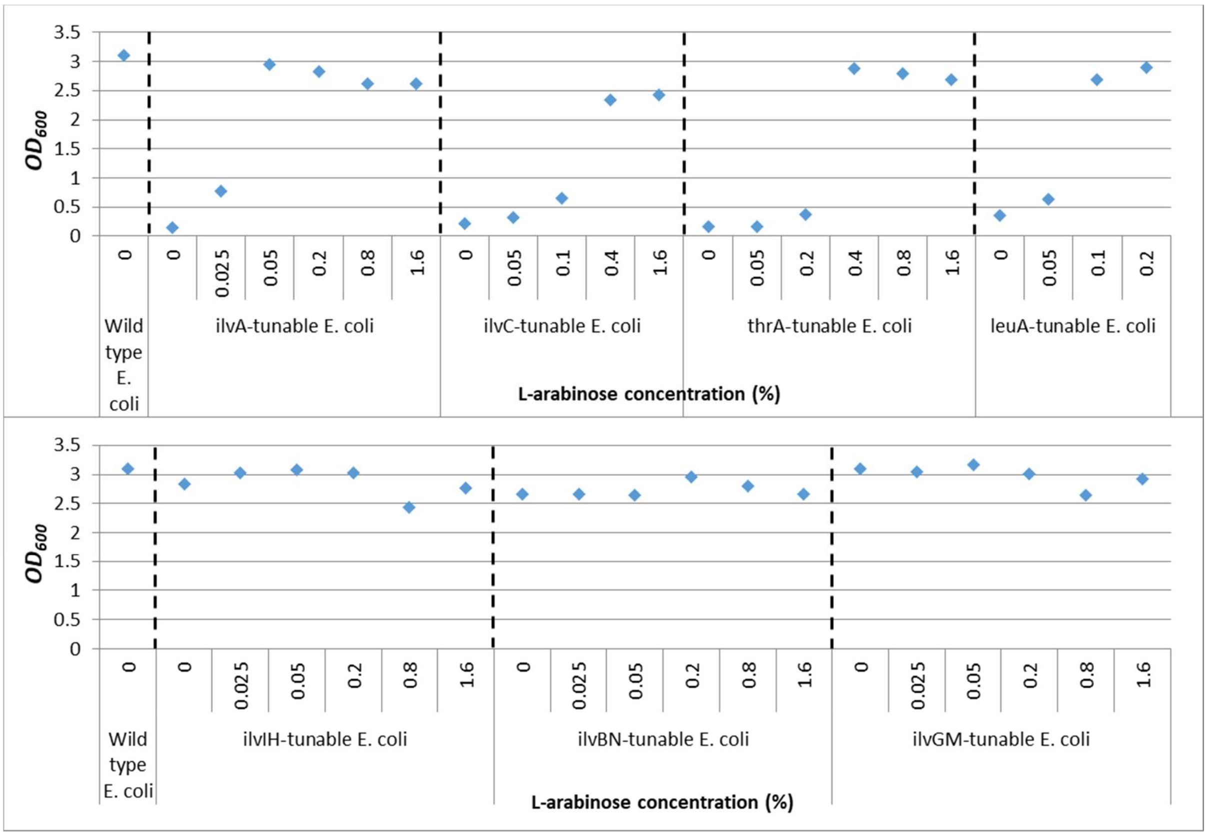

Figure S3. OD_{600} measured 16h after cultivation of *E. coli* mutant strains under different L-arabinose concentrations. The reference strain *E. coli* K-12 BW25113 pSW3_*lacI*+ is also included as a control for comparison.

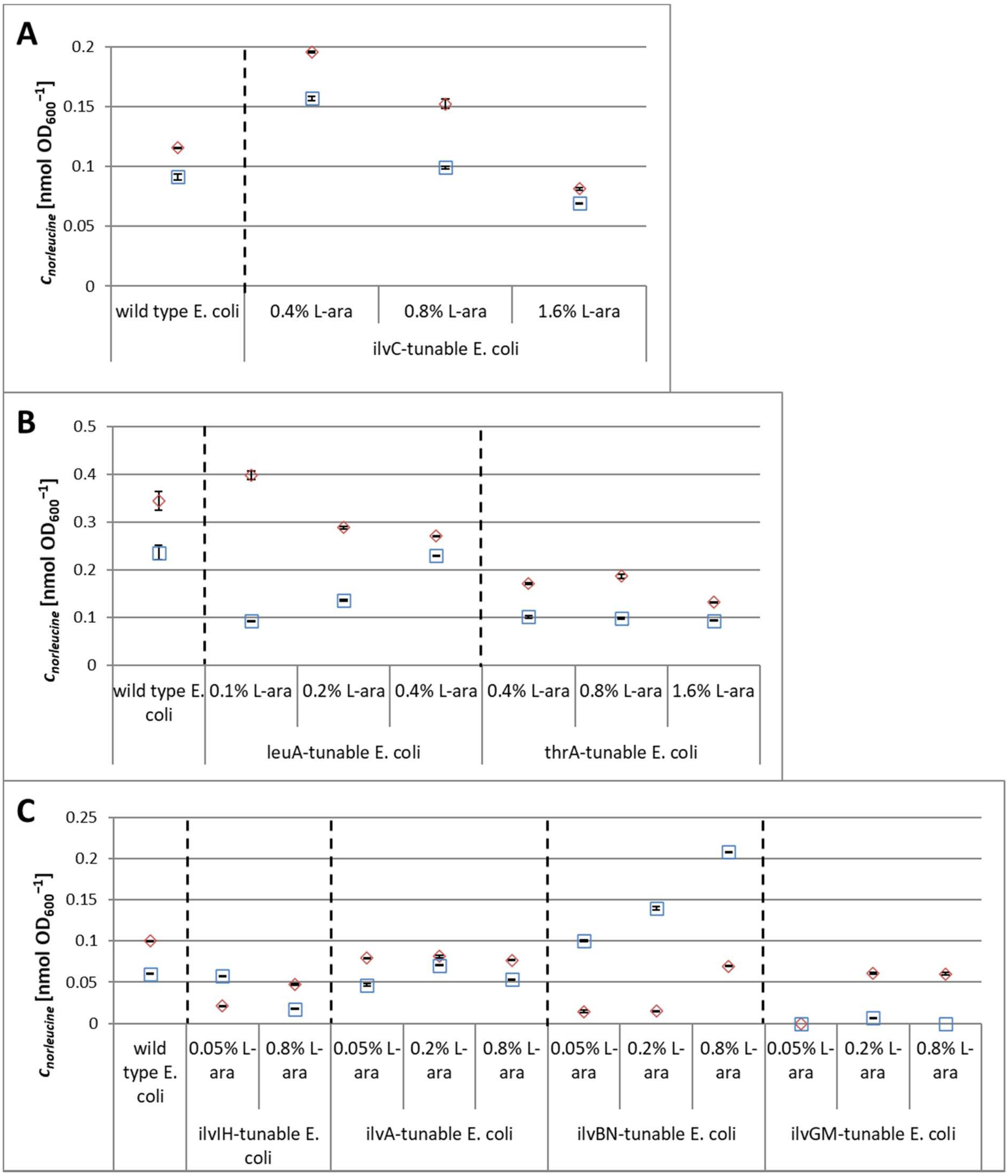

Figure S4. Molar concentrations of norleucine normalized to OD_{600} in the inclusion body fraction from samples taken from glucose-limited fed-batch cultivations 2 h after IPTG induction of *ilvC* tunable *E. coli* (A), 3.5 h after IPTG induction of *leuA* and *thrA* tunable *E. coli* strains (B) and 3 h after induction of *ilvIH*, *ilvA*, *ilvBN* and *ilvGM* tunable *E. coli* strains (C) in a 10 mL PALL24 mini-reactor with different L-arabinose concentrations and cultivation modes. Two cultivation modes were tested: reference cultivation (□) and cultivation under simultaneous pyruvate pulsing and dissolved oxygen (DO) limitation (◇). Strain *E. coli* K-12 BW25113 pSW3_*lacI*$^+$ (named "wild type *E. coli*" in the chart) was employed as the reference strain. Results represent the average of 3 technical replicates.

Table S1. Overview of the different cultivation conditions tested in each well of the first mini-reactor plate with the reference strain *E. coli* K-12 BW25113 pSW3_*lacI*$^+$, and the tunable strains for *leuA, ilvC* and *thrA.*

	1	2	3	4	5	6
A	*E. coli* K-12<br>BW25113<br>pSW3_*lacI*$^+$<br>0 % L-ara<br>Reference	*E. coli* K-12<br>BW25113<br>pSW3_*lacI*$^+$<br>0 % L-ara<br>Reference	*E. coli* K-12<br>BW25113<br>pSW3_*lacI*$^+$<br>0 % L-ara<br>Reference	*E. coli* K-12<br>BW25113<br>pSW3_*lacI*$^+$<br>0 % L-ara<br>Pyruvate + DO lim.	*E. coli* K-12<br>BW25113<br>pSW3_*lacI*$^+$<br>0 % L-ara<br>Pyruvate + DO lim.	*E. coli* K-12<br>BW25113<br>pSW3_*lacI*$^+$<br>0 % L-ara<br>Pyruvate + DO lim.
B	*leuA* tunable *E. coli*<br>0.1 % L-ara<br>Reference	*leuA* tunable *E. coli*<br>0.2 % L-ara<br>Reference	*leuA* tunable *E. coli*<br>0.4 % L-ara<br>Reference	*leuA* tunable *E. coli*<br>0.1 % L-ara<br>Pyruvate + DO lim.	*leuA* tunable *E. coli*<br>0.2 % L-ara<br>Pyruvate + DO lim.	*leuA* tunable *E. coli*<br>0.4 % L-ara<br>Pyruvate + DO lim.
C	*ilvC* tunable *E. coli*<br>0.4 % L-ara<br>Reference	*ilvC* tunable *E. coli*<br>0.8 % L-ara<br>Reference	*ilvC* tunable *E. coli*<br>1.6 % L-ara<br>Reference	*ilvC* tunable *E. coli*<br>0.4 % L-ara<br>Pyruvate + DO$_2$ lim.	*ilvC* tunable *E. coli*<br>0.8 % L-ara<br>Pyruvate + DO lim.	*ilvC* tunable *E. coli*<br>1.6 % L-ara<br>Pyruvate + DO lim.
D	*thrA tunable E. coli*<br>0.4 % L-ara<br>Reference	*thrA tunable E. coli*<br>0.8 % L-ara<br>Reference	*thrA tunable E. coli*<br>1.6 % L-ara<br>Reference	*thrA tunable E. coli*<br>0.4 % L-ara<br>Pyruvate + DO lim.	*thrA tunable E. coli*<br>0.8 % L-ara<br>Pyruvate + DO lim.	*thrA tunable E. coli*<br>1.6 % L-ara<br>Pyruvate + DO lim.

Table S22. Overview of the different cultivations conditions tested in each well of the second mini-reactor plate with the reference strain *E. coli* BW25113 pSW3_*lacI+*, and the tunalbe strains for *ilvIH, ilvA, ilvBN* and *ilvGM*.

	1	2	3	4	5	6
A	*E. coli* K-12 BW25113<br>pSW3_*lacI*$^+$<br>0 % L-ara<br>Reference	*E. coli* K-12 BW25113<br>pSW3_*lacI*$^+$<br>0 % L-ara<br>Pyruvate + DO lim.	*ilvIH* tunable *E. coli*<br>0.05 % L-ara<br>Reference	*ilvIH* tunable *E. coli*<br>0.8 % L-ara<br>Reference	*ilvIH* tunable *E. coli*<br>0.05 % L-ara<br>Pyruvate + DO lim.	*ilvIH* tunable *E. coli*<br>0.8 % L-ara<br>Pyruvate + DO lim.
B	*ilvA* tunable *E. coli*<br>0.05 % L-ara<br>Reference	*ilvA* tunable *E. coli*<br>0.2 % L-ara<br>Reference	*ilvA* tunable *E. coli*<br>0.8 % L-ara<br>Reference	*ilvA* tunable *E. coli*<br>0.05 % L-ara<br>Pyruvate + DO lim.	*ilvA* tunable *E. coli*<br>0.2 % L-ara<br>Pyruvate + DO lim.	*ilvA* tunable *E. coli*<br>0.8 % L-ara<br>Pyruvate + DO lim.
C	*ilvBN* tunable *E. coli*<br>0.05 % L-ara<br>Reference	*ilvBN* tunable *E. coli*<br>0.2 % L-ara<br>Reference	*ilvBN* tunable *E. coli*<br>0.8 % L-ara<br>Reference	*ilvBN* tunable *E. coli*<br>0.05 % L-ara<br>Pyruvate + DO lim.	*ilvBN* tunable *E. coli*<br>0.2 % L-ara<br>Pyruvate + DO lim.	*ilvBN* tunable *E. coli*<br>0.8 % L-ara<br>Pyruvate + DO lim.
D	*ilvGM* tunable *E. coli*<br>0.05 % L-ara<br>Reference	*ilvGM* tunable *E. coli*<br>0.2 % L-ara<br>Reference	*ilvGM* tunable *E. coli*<br>0.8 % L-ara<br>Reference	*ilvGM* tunable *E. coli*<br>0.05 % L-ara<br>Pyruvate + DO lim.	*ilvGM* tunable *E. coli*<br>0.2 % L-ara<br>Pyruvate + DO lim.	*ilvGM* tunable *E. coli*<br>0.8 % L-ara<br>Pyruvate + DO lim.
